# Safety and efficacy of biliary stenting combined with iodine-125 seed strand followed by hepatic artery infusion chemotherapy plus lenvatinib with PD-1 inhibitor for the treatment of extrahepatic cholangiocarcinoma with malignant obstructive jaundice

**DOI:** 10.3389/fimmu.2023.1286771

**Published:** 2024-01-15

**Authors:** Long-Wang Lin, Kun Ke, Rong Chen, Wei-Zhu Yang, Ning Huang, Zheng-Zhong Wu

**Affiliations:** Department of Interventional Radiology, Fujian Medical University Union Hospital, Fuzhou, China

**Keywords:** extrahepatic cholangiocarcinoma, hepatic artery infusion chemotherapy, iodine-125 seed strand, lenvatinib, programmed death-1 inhibitor

## Abstract

**Objectives:**

To evaluate the efficacy and safety of biliary stenting implantation with iodine-125 seed strand (SI) followed by hepatic artery infusion chemotherapy (HAIC) plus lenvatinib (Len) with programmed death-1 (PD-1) inhibitor for patients diagnosed with extrahepatic cholangiocarcinoma (ECC) and malignant obstructive jaundice (MOJ).

**Methods:**

In this single-center retrospective study, the data of ECC patients with MOJ from March 2015 to January 2023 was assessed. Using probability score matching (PSM), the selection bias of patients was reduced. Primary study outcomes included overall survival (OS) and progression-free survival (PFS). The OS and PFS were performed using the Kaplan–Meier method and evaluated with the log-rank test.

**Results:**

A total of 104 patients were enrolled finally, including 52 patients treated with interventional therapy (SI+HAIC) plus Len with PD-1 inhibitor (SI+HAIC+Len+P group) and 52 patients treated with interventional therapy (SI+HAIC) plus lenvatinib (SI+HAIC+Len group). 26 pairs of patients were matched after PSM analysis. After PSM analysis, the median OS and PFS in the SI+HAIC+Len+P group were significantly longer compared to those in the SI+HAIC+Len group (OS:16.6 *vs.* 12.3 months, *P* = 0.001; PFS:12.6 *vs* 8.5 months, *P* = 0.004). The DCR was significantly different between groups (*P* = 0.039), while ORR not (*P* = 0.548). The addition of PD-1 inhibitor was generally well tolerated without treatment-associated mortality.

**Conclusion:**

Interventional therapy (SI+HAIC) plus Len with PD-1 inhibitor was effective for ECC patients accompanied by MOJ with a manageable safety profile.

## Introduction

Extrahepatic cholangiocarcinoma (ECC) is a highly aggressive malignancy that affects the biliary duct system (1). The prognosis of advanced ECC, especially in patients with malignant obstructive jaundice (MOJ), is notably unfavorable due to the occurrence of biliary tract occlusion, which can cause abnormal liver function, poor nutritional absorption, and low protein levels, subsequently leading to a series of aftereffects (2–4). The previous studies had substantiated that the therapeutic approach for ECC patients with MOJ involves two key elements: first, restoring biliary tract flow, and second, aiming to inhibit the progression of biliary tract tumor. ECC with MOJ requires the removal of biliary blockage, improving liver function, and relieving symptoms before further treatments (5, 6). Biliary stenting combined with iodine-125 seed strand (SI) has become the preferred treatment method (7). Radioactive iodine-125 seed brachytherapy is a minimally invasive treatment utilizing advanced imaging technology. The procedure involves the permanent or temporary implantation of iodine-125 directly into the tumor, enabling the continuous emission of γ-rays (8). This sustained release of radiation effectively contributes to the eradication of the tumor. The seed strand consists of iodine-125 seeds that are inserted into a catheter to create regular and linear arrangements. These seed strands are then positioned within the biliary duct using catheter and guidewire technology (9). The utilization of seed strands offers several benefits, including sustained low-dose radiation, significant local dose accumulation, straightforward operation, convenient protection measures, reliable repeatability, and cost-effectiveness. A multicenter phase III clinical trial demonstrated that the combination of SI therapy may enhance the patency and survival prognosis of patients with unresectable ECC with MOJ (10). This improvement can be attributed to the physical expansion of stents within the stenotic segment, as well as the continuous irradiation of the seeds to inhibit tumor proliferation and growth in the biliary tract (11, 12). Consequently, long-term obstructive jaundice is alleviated, leading to improved liver function and extended survival time for patients (13). Moreover, a study conducted by Wu et al. revealed that SI therapy and hepatic artery infusion chemotherapy (HAIC) prolonged overall survival (OS) to 12 months (14). This combined treatment approach effectively reduced bilirubin levels and diminished tumor lesions. Nevertheless, the efficacy of combination therapy for patients with ECC did not demonstrate a highly satisfactory outcome.

More recently, for patients with advanced cholangiocarcinoma, lenvatinib (Len) plus programmed death-1 (PD-1) inhibitor therapy has been proven to be encouraging and has shown promising clinical results (15). The combination of Len plus PD-1 inhibitor therapy has been evaluated in previous studies in cholangiocarcinoma with an objective response rate (ORR) of 10-25%, a median OS of 7.4–11.0 months, and progression-free survival (PFS) of 1.8–4.9 months (15–17). However, in the case of patients with ECC with MOJ, the presence of biliary tract occlusion leading to impaired liver function has rendered the administration of Len and PD-1 inhibitors a relative contraindication, thereby restricting their utilization.

Based on the aforementioned findings, interventional therapy (SI+HAIC) alleviated obstructive jaundice rapidly and improved liver function, making ECC patients with MOJ eligible for Len plus PD-1 inhibitor therapy. Therefore, whether interventional therapy (SI+HAIC) combined with Len and PD-1 inhibitor could provide longer OS and a better tumor response rate for ECC patients with MOJ. It is hypothesized that this triple therapy might show potential synergistic effects and provide a significant survival benefit for ECC patients with MOJ.

## Materials and methods

### Patients

**Figure 1:**
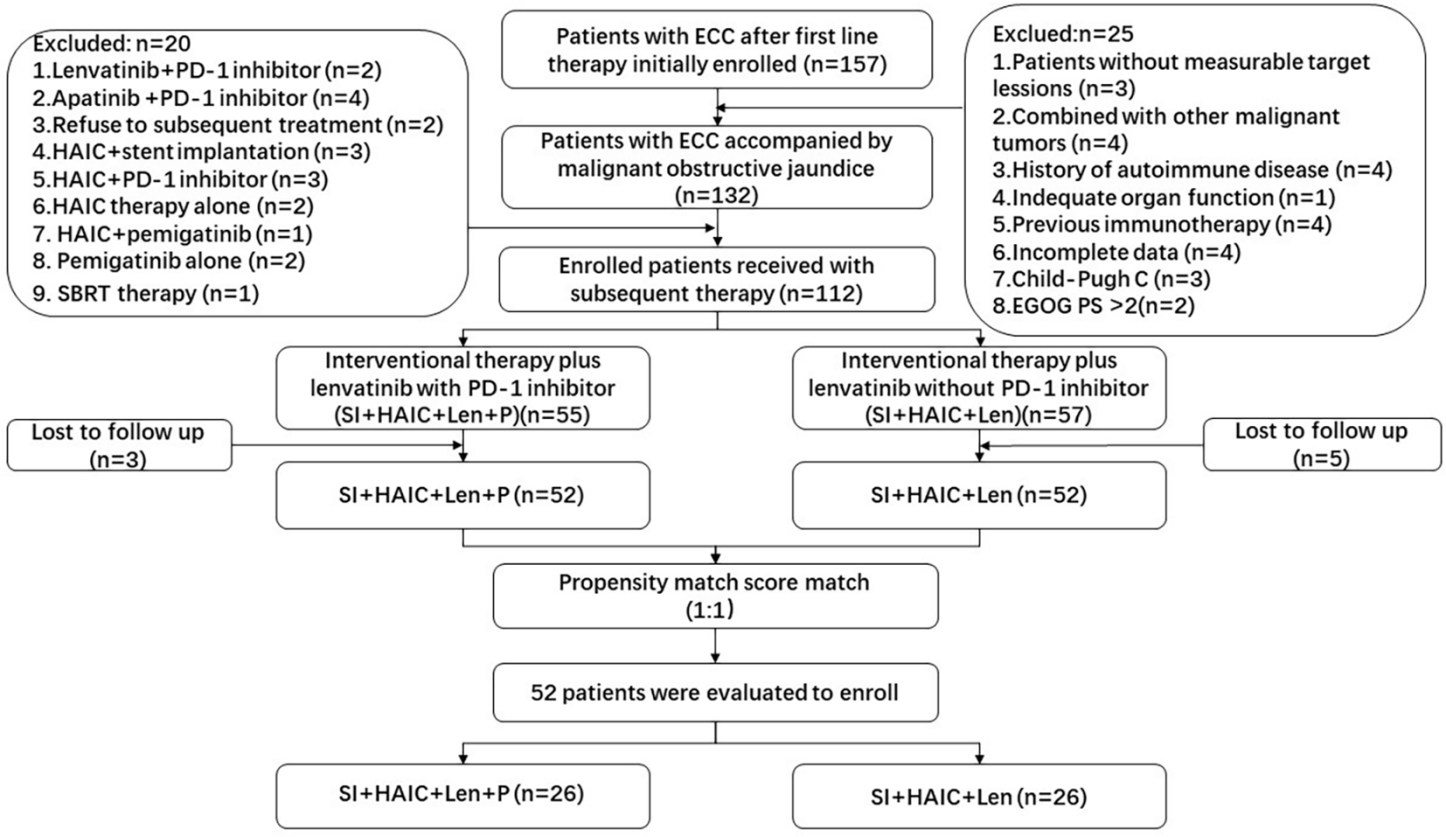
Flowchart of the patient selection process. ECC, extrahepatic cholangiocarcinoma; SBRT, stereotactic body radiotherapy; PD-1, programmed death-1; HAIC, hepatic artery infusion chemotherapy; ECOG-PS, Eastern Cooperative Oncology Group performance status.

This retrospective study was approved by the Ethics Committee in compliance with the principles outlined in the Declaration of Helsinki. Given the retrospective and anonymous nature of the study, the requirement for informed consent was waived. 157 ECC patients were initially evaluated from March 2015 to January 2023 (**Figure 1**). Finally, 104 ECC patients with MOJ were enrolled in this study, stratified as 52 patients treated with interventional therapy (SI+HAIC) plus Len with PD-1 inhibitor (SI+HAIC+Len+P group), and 52 patients treated with interventional therapy (SI+HAIC) plus Len (SI+HAIC+Len group).

The inclusion criteria were: 1) age ≥ 18 and ≤ 75 years; 2) unresectable ECC patients accompanied by MOJ; 3) pathologic histology confirms the diagnosis of ECC by biopsy; 4) symptoms such as jaundice relate to biliary obstruction; 5) Eastern Co-operative Oncology Group Performance Status (ECOG-PS) score ≤ 2; and 6) Child-Pugh class A or B. Unresectable ECC should meet the following criteria of unresectability (18), for hilar cholangiocarcinoma, including distant metastatic disease (nonsatellite hepatic metastsses, lymph node metastases and distant metastases in other organ/sites), extensive local involvement of portal, hepatic artery and second biliary radicals, and inadequate future liver remnant(< 30% future liver remnant in patients with normal hepatic parenchyma and < 2 contiguous segments with adequate portal venous and hepatic arterial inflow adequate hepatic venous drainage, and adequate biliary drainage); for distal cholangiocarcinoma, including distant metastatic disease (distant metastases liver or other organs and lymph node metastases) and major vascular involvement of significant portal vein, superior mesenteric artery, and common or proper hepatic artery. respectively, MOJ should meet the following criteria (19), including clinical symptoms consistent with a diagnosis of biliary obstruction with jaundice, computed tomography (CT) or magnetic resonance imaging (MRI) indicating obstructive jaundice and a diagnosis of malignant pathology.

The exclusion criteria were: 1) age < 18 or > 75 years; 2) patients receiving previous immunotherapy; 3) patients receiving other tyrosine kinase inhibitors (TKI) therapy; 4) patients with severe autoimmune diseases; 5) ECOG-PS score > 2; 6) patients receiving other subsequent treatments; 7) Child-Pugh class C; 8) patients with incomplete data; 9) patients lost to follow-up; and 9) contraindications for HAIC, Len, PD-1 inhibitor.

### Materials and devices

1. Each iodine-125 seed (Beijing ZHIBO Bio-Medical Technology Company, China) was measured at 0.8 mm in diameter and 4.5 mm in length. Each seed had a radioactivity of 0.7 mCi, a radiation energy of 27.4 KeV, and a 59.6-day half-life.
2. The biliary self-expanding metal stent SMART Control (Cordis, Johnson & Johnson, USA) was placed with a length of 60–100 mm and a diameter of 6–8 mm.
3. 8F biliary drainage tube (Argon Medical Devices, Plano, TX, USA) and a device (TPS; BT-RSI; Yuan Bo, Beijing, China) that implanted iodine-125 seeds were also used.

### The production process of iodine-125 seed strand

Utilizing the TPS, we devised preoperative seed implantation strategies by computing the overall activity and seed demand. The precise longitudinal dimension of the implanted seeds was determined through the application of the subsequent formula: occluded segment length (mm) divided by 4.5. The quantity of seeds was adjusted in accordance with the patient’s condition, either increasing or decreasing as necessary. To ensure sufficient drainage, it was imperative to seal and thermally treat the distal end of the 4F tube within the 8F biliary drainage tube. Throughout the procedure, each seed was individually inserted into the tube, with a gelatin sponge strip employed to seal the proximal lumen and secure the seeds in place.

### Biliary stent implantation

**Figure 2:**
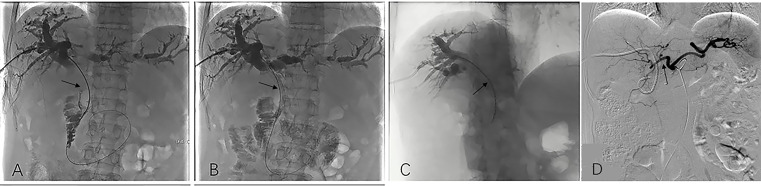
Image dates image showed the operation procedure of biliary stenting with iodine-125 seed strand placed in the obstructed segment of the bile duct. Imaging under donor-specific antibody (DSA) showed a bile duct tumor thrombus and a filling defect inside the bile duct (black arrow) **(A)**; DSA imaging showed biliary stenting were placed in the obstructed segment from the hilar region to the distal common bile (black arrow). Subsequently bile duct drainage restored to open and the bile duct filling defect is disappeared **(B)**. DSA Imaging showed iodine-125 radioactive seed strips were placed through the PTCD tube (black arrow) **(C)**; After the total serum bilirubin decreased to normal and the liver function of the patient recovered, HAIC was conducted (black arrow) **(D)**. HAIC, hepatic artery infusion; PTCD, percutaneous transhepatic cholangial drainage.

Cholangiography was employed to ascertain the extent of the stent. A 0.035-inch exchange guidewire was introduced at the point of entry into either the duodenum or the distal end of the common bile duct. Subsequently, utilizing an elongated sheath, the stent system was inserted in such a manner that the designated markers at both termini were positioned at a minimum distance of one centimeter above the obstructed segment, after which the stent was released (**Figures 2A, B**). To validate the efficacy, cholangiography was once again employed.

### Iodine-125 seed strand implantation

The administration of the iodine-125 seed strand into the biliary tract was performed via an 8F biliary drainage tube. Subsequently, the seed strand and drainage tube were connected using a 3-way tube to enable continued bile drainage. Following the release of the stent, the seed strand was promptly positioned within the stenosis segment. As the guidewire was retracted, the seed strand was released in the bile duct, facilitating the irradiation of the seeds onto the obstruction (**Figure 2C**).

### HAIC

The HAIC procedure was performed by experienced physicians following the implantation of biliary stenting with iodine-125 seed strand. This was done at a three-week interval. The modified Seldinger’s technique was employed after preparing the skin and administering local anesthesia, whereby the right femoral artery was punctured using a vascular sheath. The catheter was placed in the common hepatic artery, which served as the target artery for perfusion chemotherapy (**Figure 2D**). Chemotherapy agent was infused through the microcatheter as follows: gemcitabine is administered over 30 minutes at a dose of 600-1000 mg/m^2^, and oxaliplatin is administered over 2 hours at a dose of 60-100 mg/m^2^.

### Lenvatinib and PD-1 inhibitor

Within 5-7 days of HAIC, the dose of Len was given orally in either a form of 12 mg (for patients weighing ≥ 60 kg) or 8 mg (for patients weighing < 60 kg), If patients developed grade 3 or 4 TRAEs, the Len dosage was adjusted to 8 mg (for patients weighing ≥ 60 kg) or 4 mg (for patients weighing < 60 kg). Within 5-7 days of HAIC, simultaneous administration of PD-1 inhibitors was conducted. Tislelizumab or sintilimab were given at a dosage of 200 mg for every 3 weeks. When grade 3 or 4 TRAEs occurred, corticosteroids were used.

Len and PD-1 inhibitors were discontinued in the case of grade 3 or 4 treatment-related adverse events (TRAEs) continuing after adjustment. Additionally, according to the investigator’s discretion, as soon as the patient was able to tolerate the dosage or the toxicity diminished, the dose was recovered.

### Treatment evaluation and follow-up

**Figure 3:**
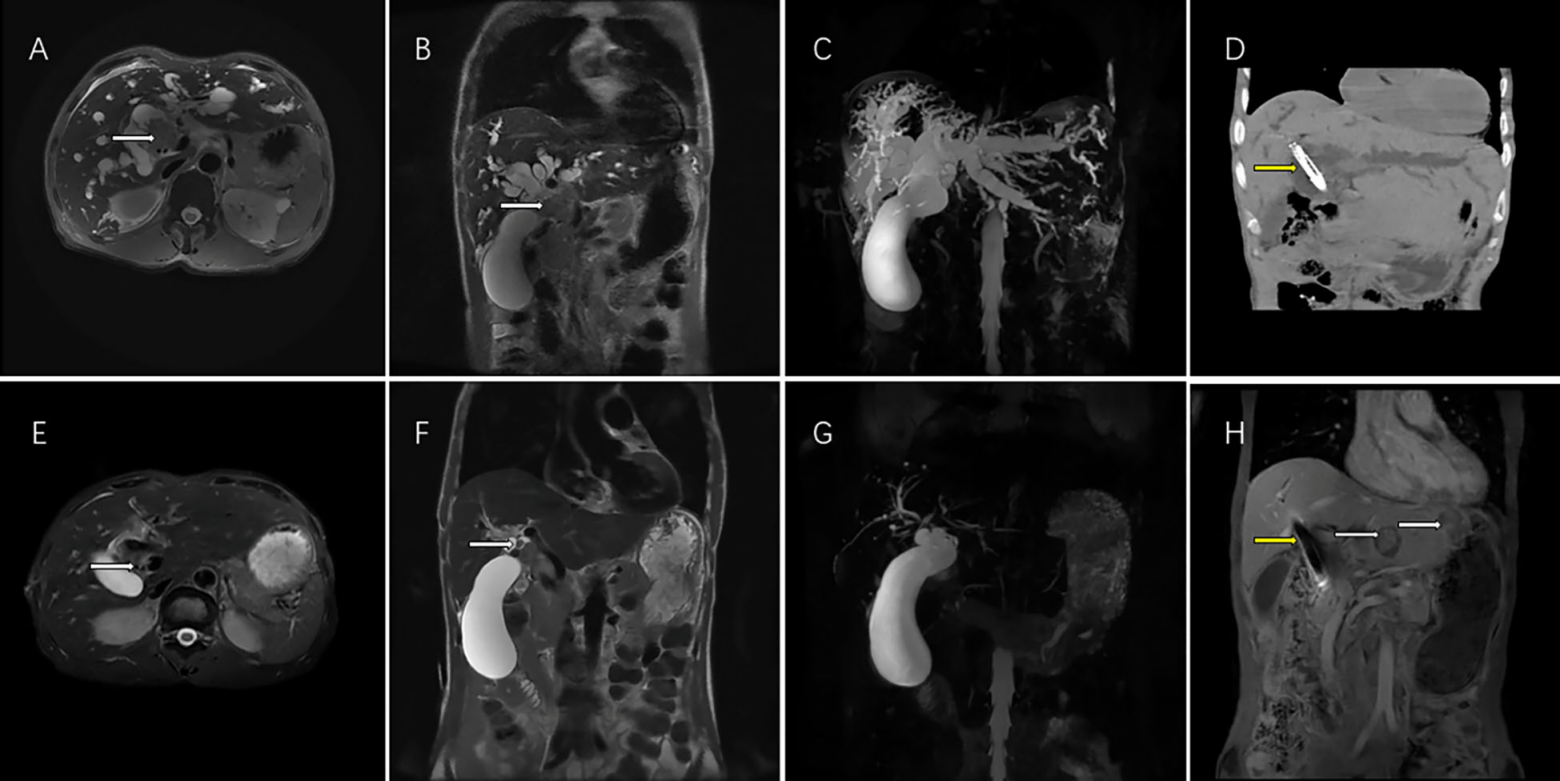
This magnetic resonance (MR) images showed the imaging feature of 62-year-old men with perihilar cholangiocarcinoma accompanied by malignant obstructive jaundice. T2 weighted cross sectional imaging **(A)** and T2 weighted coronal imaging **(B)** showed the bile duct tumor of the hilar region (white arrow) with bile duct dilatation before interventional treatment plus lenvatinib with PD-1inbhibitor therapy; Magnetic resonance cholangial pancreatography (MRCP) showed thick intrahepatic dilated bile ducts and a large gallbladder **(C)**; Computed tomography showed biliary stenting with iodine-125 seed strand (yellow arrow) were in good position 1 month after the operation **(D)**. T2 weighted cross sectional imaging **(E)**, T2 weighted coronal imaging **(F)** showed that the bile duct tumor volumes of the hilar region shrank (white arrow), the bile duct returned to normal size and achieved PR according to the mRECIST criteria after 5 courses of interventional treatment plus lenvatinib plus PD-1inbhibitor therapy. MRCP showed the normalization of the intrahepatic bile ducts after treatment **(G)**. Although hepatic portal tumor was well controlled (yellow arrow), two new intrahepatic metastatic lesions appeared (yellow arrow) in the left liver 12 months after interventional treatment plus lenvatinib with PD-1inhibitor therapy and achieved PD according to the mRECIST criteria **(H)**. PR, partial response; mRECIST, the Modified Response Evaluation Criteria in Solid Tumors. PD, progression disease; mRECIST, the Modified Response Evaluation Criteria in Solid Tumors; PD-1, programmed death-1.

The primary outcomes were OS and PFS. The OS was defined as the period from the stent implantation to the patient’s death, while PFS was defined as the period from stent implantation until the patient’s first documented progression of disease or death. Additionally, treatment response was determined using the Modified Response Evaluation Criteria in Solid Tumors (mRECIST) criteria which included complete response (CR), partial response (PR) (**Figures 3A–G**), stable disease (SD), and progression disease (PD) (**Figure 3H**). ORR was defined as the sum of CR and PR, while the disease control rate (DCR) was defined as the sum of CR and PR. Every five to seven weeks, imaging examinations (CT or MRI) were performed on patients to assess their disease status. The Common Terminology Criteria for Adverse Events version 5.0. was used to evaluate TRAEs.

### Statistical analysis

Prism 8 (GraphPad Software, San Diego, CA, USA) and SPSS 25.0 (IBM, Chicago, IL, USA) were used for all statistical analyses. Continuous variables were displayed using the mean with standard deviation (SD) (mean ± SD) or median (interquartile range) according to normal distribution., while categorical variables were displayed utilizing numbers and percentages (n, (%)). The independent sample t-test or Mann–Whitney U test was used to compare continuous variables based on normal distribution, while the chi-square test was used to compare categorical variables. Using the Kaplan-Meier method, the survival curves were analyzed, and log-rank tests were used to determine differences. Univariate and multivariate analyses for OS and PFS were conducted using Cox proportional hazards models. the univariate Cox proportional hazards model was applied to each variable, followed by fitting the variables with a two-sided P value < 0.05 to the multivariate model. To determine their value as independent predictors of OS and PFS, statistical significance was identified at P value < 0.05.

The probability score matching (PSM) analysis was used to reduce bias in patient selection between two groups. Our model matched variables that showed significant differences between patients or associations with patient selection. With one-to-one matching but no replacement, the caliper’s value was 0.02.

## Results

### Baseline characteristics

Between March 2015 and January 2023, a total of 104 patients (52 in the SI-HAIC-Len-P group and 52 in the SI-HAIC-Len group) were ultimately included in the study. Following the PSM analysis, 26 pairs of patients were successfully matched. The detailed baseline characteristics are presented in **Table 1**. Notably, no statistically significant differences were observed between the two groups, both before and after the PSM procedure (*P* > 0.05). Each patient underwent the technique successfully. After PSM, the average number of iodine-125 seeds loaded was 15.4 ± 5.1 (range, 10-28) in the SI+HAIC+Len+P group compared to 16.3 ± 4.4 (range, 8-32) in the SI+HAIC+Len group (*P* = 0.657), respectively, the iodine-125 seeds strand did not exhibit signs of displacement in the MR and CT images. A total of 206 HAIC procedures were conducted, with 104 performed in the SI+HAIC+Len+P groups and 102 in the SI+HAIC+Len group; The mean number of HAIC procedures in the SI+HAIC+Len+P groups was 4.0 ± 1.4 (range 2-6) compared to 3.9 ± 1.3 (range 2-6) in the SI+HAIC+Len group, with no statistically significant difference (*P* = 1.000). Within the SI+HAIC+Len+P group, two types of PD-1 inhibitors, namely sintilimab (n = 5, 19.24%) and tislelizumab (n = 21, 80.76%), were administered for an average of 8 cycles (ranging from 2 to 12).

**Table 1:** Baseline characteristics of patients.

Characteristics	Before PSM SI+HAIC+Len+P (n=52)	SI+HAIC+Len (n=52)	*P value*	After PSM SI+HAIC+Len+P (n=26)	SI+HAIC+Len (n=26)	*P value*
Age (years)	64.4 ± 11.1	66.6 ± 10.5	0.453	67.1 ± 10.5	64.3 ± 9.8	0.496
Sex, *n (*%)			1.000			0.695
Male	34(65.38%)	34(65.38%)		12(46.15%)	14(53.84%)	
Female	18(34.62%)	18(34.62%)		14(53.85%)	12(46.16%)	
Etiology, *n* (%)			0.734			1.000
Hepatitis B	12(23.08%)	10(19.24%)		4(15.38%)	5(19.23%)	
Hepatitis C	0(0.00%)	0(0.00%)		0(0.00%)	0(0.00%)	
Nonhepatitis B and C	40(76.92%)	42(80.76%)		22(84.62%)	21(80.77%)	
Child Pugh score, *n* (%)			0.425			0.695
5-6	22(42.30%)	28(70.27%)		14(53.85%)	12(46.16%)	
7-9	30(57.70%)	24(29.73%)		12(46.15%)	14(53.84%)	
TNM stage			0.780			0.695
III	28(53.84%)	30(57.69%)		14(53.85%)	12(46.16%)	
IV	24(46.16%)	22(42.31%)		12(46.15%)	14(53.85%)	
Tumor subtype, n (%)			0.695			0.578
Perihilar cholangiocacinoma	28(53.84%)	26(50.00%)		13(50.00%)	11(42.30%)	
Distal cholangiocacinoma	24(46.16%)	26(50.00%)		13(50.00%)	15(57.70%)	
Differentiated histology, n (%)			0.843			
Poorly	24(46.15%)	20(38.46%)		12(46.15%)	10(38.46%)	0.386
Moderately	20(38.46%)	22(42.31%)		8(30.76%)	14(53.84%)	
Well	8(15.38%)	10(19.24%)		6(23.07%)	2(7.70%)	
ECOG performance score, *n* (%)			0.397			0.691
0-1	34(65.38%)	28(53.84%)		16(61.53%)	14(53.84%)	
1-2	18(34.62%)	24(46.16%)		10(38.47%)	12(46.16%)	
Largest tumor size (cm)			0.375			1.000
>3	14(26.92%)	20(38.46%)		10(38.46%)	10(38.46%)	
≤3	38(73.08%)	32(51.54%)		16(61.54%)	16(61.54%)	
CA199 (U/mL)			0.587			0.781
>200	30(57.69%)	26(50.00%)		14(53.85%)	13(50.00%)	
≤200	22(42.31%)	26(50.00%)		12(46.15%)	13(50.00%)	
Prior chemotherapy cycles						0.569
≤6	24(46.15%)	22(42.31%)	0.693	11(42.30%)	9(34.61%)	
>6	28(53.85%)	30(57.69%)	15(57.70%)	17(65.39%)	
Extrahepatic metastases, *n* (%)			0.337			0.658
Present	10(19.23%)	16(30.76%)		8(30.76%)	6(23.07%)	
Absent	42(80.77%)	36(69.24%)		18(69.24%)	20(76.93%)	
Ascites			0.248			1.000
Present	4(7.69%)	12(23.07%)		4(15.38%)	4(15.38%)	
Absent	48(92.31%)	40(76.93%)		22(84.62%)	22(84.62%)	
Platelet (10^9^/L)	233.27 ± 96.02	202.77 ± 77.21	0.213	236.4 ± 93.6	206.1 ± 97.7	0.426
WBC (10^9^/L)	7.60 ± 2.36	7.73 ± 2.41	0.854	7.1 ± 2.8	7.7 ± 2.7	0.560
Neutrophils (10^9^/L)	7.73 ± 2.41	5.73 ± 2.72	0.854	5.4 ± 3.1	4.7 ± 1.6	0.451
Lymphocyte (10^9^/L)	1.10(0.76-1.49)	0.85(0.66-1.30)	0.198	1.00(0.76-1.30)	0.86(0.71-1.30)	0.884
TBIL (umol/L) Before treatment	249.5 ± 50.5	257.4 ± 48.4	0.416	265.4 ± 41.7	244.73.8 ± 6	0.174
2 weeks after treatment	48.9 ± 12.4	49.6 ± 13.4	0.783	51.3 ± 11.9	45.3 ± 14.2	0.110
4 weeks after treatment	21.6 ± 5.4	22.7 ± 4.6	0.634	22.4 ± 4.8	21.6 ± 5.2	0.823
ALB (g/L)	31.1 ± 4.7	32.2 ± 4.9	0.381	30.1 ± 5.2	31.3 ± 4.3	0.538
ALT (IU/L)	43(28-78)	42(26-69)	0.491	38(24-49)	39(30-53)	0.399
AST (IU/L)	43(29-74)	50(37-50)	0.336	36(24-58)	49(30-60)	0.115
GGT (IU/L)	243.4 ± 176.7	250.6 ± 194.9	0.890	165.6 ± 71.9	250.4 ± 202.8	0.168
ALP (IU/L)	311.6 ± 218.4	365.3 ± 279.5	0.444	281.8 ± 220.3	418.8 ± 346.3	0.241
Radiotherapy dose (iodine-125 number)	16.6 ± 5.3	16.5 ± 5.9	0.942	15.4 ± 5.1	16.3 ± 4.4	0.657
HAIC courses	4.0 ± 1.5	3.8 ± 1.5	0.982	4.0 ± 1.4	3.9 ± 1.3	1.000
2	10(19.23%)	12(23.07%)		4(15.38%)	4(15.38%)	
3	12(23.07%)	12(23.07%)		6(23.07%)	7(26.92%)	
4	20(38.46%)	18(34.61%)		11(42.30%)	9(34.61%)	
5	6(11.53%)	5(9.61%)		2(7.68%)	3(11.53%)	
6	4(7.69%)	5(9.61%)		3(11.53%)	3(11.53%)	

Data are presented as mean ± SD, median (interquartile range),and n (%); HAIC, hepatic artery infusion chemotherapy; ECOG, Eastern Cooperative Oncology Group; Len, Lenvatinib; TNM, tumor node metastasis; CA199, carbohydrate antigen 199; WBC, white blood cell; TBIL, total bilirubin; GGT, γ-glutamyl transferase; ALP, alkaline phosphatase; ALB, albumin; AST, aspartate aminotransferase; ALT, alanine aminotransferase.

### Treatment response

According to the mRECIST criteria, the ORR and DCR were both evaluated to determine the tumor response, which were shown in **Table 2**. Before PSM analysis, the DCR in the SI+HAIC+Len+P group was significantly longer compared to the SI+HAIC+Len group (78.85% *vs.* 59.62%, *P* = 0.034). After PSM analysis, the DCR in the SI+HAIC+Len+P group was significantly higher than in the SI+HAIC+Len group (79.17% *vs*. 53.84%, *P* = 0.039). However, there were no statistically significant differences observed between the groups in terms of ORR before PSM (30.76% *vs*. 23.07%, *P* = 0.377) and after PSM (34.61% *vs.* 26.92%, *P* = 0.548).

**Table 2:** Treatment response before and after PSM analysis based on the mRECIST criteria between two groups.

Curative effect	Before PSM SI+HAIC+Len+P (n=52)	SI+HAIC+Len (n=52)	*P value*	After PSM SI+HAIC+Len+P (n=26)	SI+HAIC+Len (n=26)	*P value*
Complete response (CR)	0(0.00%)	0(0.00%)		0(0.00%)	0(0.00%)	
Partial response (PR)	16(30.76%)	12(23.07%)		9(34.61%)	7(26.92%)	
Stable disease (SD)	25(48.07%)	19(36.55%)		12(46.15%)	7(26.92%)	
Progressive disease (PD)	11(21.15%)	21(40.38%)		5(20.83%)	12(46.15%)	
Overall response rate (ORR)	16(30.76%)	12(23.07%)	0.377	9(34.61%)	7(26.29%)	0.548
Disease control rate (DCR)	41(78.85%)	31(59.62%)	0.034	21(79.17%)	14(53.84%)	0.039

HAIC, hepatic artery infusion chemotherapy; mRECIST, Modified Response Evaluation Criteria in Solid Tumors; PSM, propensity-matched.

### OS and PFS

**Figure 4:**
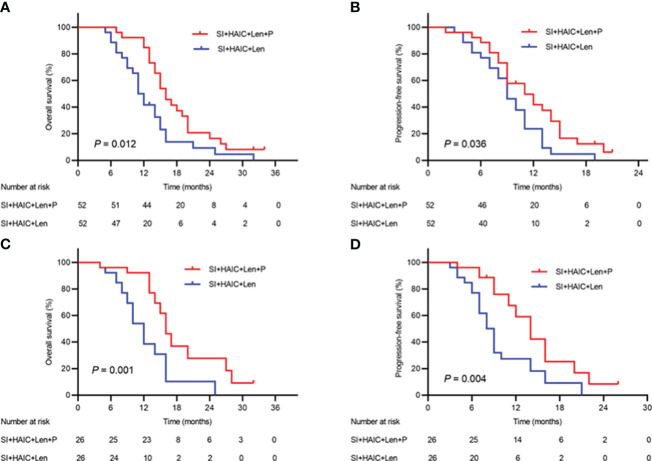
Kaplan−Meier (KM) analysis of ECC patients receiving the combination therapy of interventional treatment plus lenvatinib with or without PD-1 inhibitor. **(A)** the KM analysis of overall survival time before PSM analysis; **(B)** the KM analysis of time to progression before PSM analysis; **(C)** the KM analysis of overall survival time after PSM analysis; **(D)** the KM analysis of time to progression after PSM analysis. SI, biliary stenting implantation with iodine-125 seed strand; HAIC, hepatic artery infusion chemotherapy; PD-1, programmed death-1; ECC, extrahepatic cholangiocarcinoma; PSM, propensity-matched.

The median follow-up time was 31.8 months (range, 4.1–62.3 months). During the follow-up period, a total of 94 patients died, including 46 (88.46%) in the SI+HAIC+Len+P group and 48 (92.31%) in the SI+HAIC+Len group. Before PSM analysis, the median OS in the SI+HAIC+Len group (16.2; 95% confidence interval [CI], 14.3~17.7 months) was significantly longer compared to the SI+HAIC+Len group (11.4; 95% CI, 9.9~12.1 months) (*P* = 0.012)(**Figure 4A**); After PSM analysis, the median OS in the SI+HAIC+Len+P group (16.6; 95% CI, 14.4~17.6 months) was significantly longer compared to the SI+HAIC+Len group (12.3; 95% CI, 10.2~13.8 months) (*P* = 0.001) (**Figure 4C**).

A total of 32 patients experienced tumor progression overall, including 11 patients (21.15%) in the SI+HAIC+Len+P group and 21 patients (40.38%) in the SI+HAIC+Len group. Before PSM analysis, the median PFS in the SI+HAIC+Len+P group (11.4; 95% CI, 8.3~13.6 months) was significantly longer compared to the SI+HAIC+Len group (9.6; 95% CI, 7.9~10.1 months) (*P* = 0.036) (**Figure 4B**). After PSM analysis, the median PFS in the SI+HAIC+Len+P group (12.6; 95% CI, 11.6~14.3 months) was significantly longer compared to the SI+HAIC+Len group (8.5; 95% CI, 6.7~9.3 months) (*P* = 0.004) (**Figure 4D**).

### Factors associated with OS and PFS

Before PSM analysis, only subsequent therapy option (SI+HAIC+Len *vs.* SI+HAIC+Len+P) was independent risk factor associated with OS mortality based on the multivariate analysis (hazard ratio [HR], 2.025; 95% CI, 1.143-3.055; *P* = 0.001) (**Table 3**); Meanwhile, only subsequent therapy options (SI+HAIC+Len *vs.* SI+HAIC+Len+P) was independent risk factor associated with PFS mortality based on the multivariate analysis (HR, 1.820; 95% CI, 1.191-2.782, *P* = 0.006) (**Table 4**). After PSM analysis, multivariate Cox analysis further supported the finding that the therapy option was the only independent predictor of PFS and OS (**Tables 3**, **4**).

**Table 3:** Univariable and multivariable Cox regression analyses for time to OS before and after PSM analysis.

Characteristics	Before PSM Univariable analysis		Multivariable analysis		After PSM Univariable analysis		Multivariable analysis	
	HR (95% CI) *value*	*P*	HR (95% CI)	*P value*	HR (95% CI)	*P value*	HR (95% CI)	*P* *value*
therapy options		**0.001**		**0.001**		**0.002**		**0.002**
SI+HAIC+Len+P	reference		reference		reference		reference	
SI+HAIC+Len	2.025(1.143-3.055)		2.025(1.143-3.055)		2.684(1.436-5.019)		2.684(1.436-5.019)	
Age (years)	1.014(0.982-1.048)	0.389			0.970(0.920-1.023)	0.263		
Sex		0.383				0.233		
Male	reference				reference			
Female	0.760(0.410-1.408)				0.603(0.263-1.384)			
Etiology		0.958				0.940		
Hepatitis B	1.018(0.516-2.011)				1.164(0.368-2.525)			
Others	reference				reference			
Child Pugh score		0.862				0.890		
5-6	0.950(0.535-1.687)				0.960(0.465-2.419)			
7-9	reference				reference			
Tumor subtype		0.058				0.280		
Perihilar cholangiocacinoma	reference				reference			
Distal cholangiocacinoma	0.681(0.457-1.014)				0.863(0.574-1.243)			
Differentiated histology		0.955				0.924		
poorly	reference				reference			
others	0.989(0.677-1.446)				0.975(0.577-1.647)			
TNM stage		0.480				0.479		
III	reference				reference			
IV	1.234(0.689-2.211)				1.347(0.591-3.072)			
Prior chemotherapy cycles		0.298				0.321		
≤6	reference				reference			
*>*6	0.467(0.111-1.958)				0.471(0.107-2.081)			
Largest tumor size (cm)		0.140				0.264		
≤3	reference				reference			
*>*3	1.610(1.030-2.539)				3.393(1.616-7.125)			
CA199 (U/ml)		0.556				0.459		
>200	1.189(0.668-2.118)				1.628(0.692-3.976)			
≤200	reference				reference			
Extrahepatic metastases		0.817				0.702		
Yes	1.082(0.557-2.098)				1.238(0.339-2.072)			
No	reference				reference			
Ascites		0.522				0.624		
Yes	1.305(0.577-2.953)				1.318(0.437-3.976)			
No	reference				reference			
ECOG PS		0.851				0.553		
0-1	reference				reference			
1-2	1.060(0.577-1.946)				1.274(0.333-1.802)			

HR, hazard ratio; CI, confidence interval; ECOG, Eastern Cooperative Oncology Group; TNM, tumor node metastasis; CA199, carbohydrate antigen 199; PSM, propensity-matched; HAIC, hepatic artery infusion chemotherapy. The bold values denote statistical significance at P<0.05 level.

**Table 4:** Univariable and multivariable cox regression analyses for time to PFS before and after PSM analysis.

Characteristics	Before PSM Univariable analysis		Multivariable analysis		After PSM Univariable analysis		Multivariable analysis	
	HR (95% CI)	*P value*	HR (95% CI)	*P value*	HR (95% CI)	*P value*	HR (95% CI)	*P value*
therapy options		**0.006**		**0.006**		**0.047**		**0.047**
SI+HAIC+Len+P	reference		reference		reference		reference	
SI+HAIC+Len	1.820(1.191-2.782)		1.820(1.191-2.782)		2.372(1.011-3.562)		2.372(1.011-3.562)	
Age (years)	1.008(0.979-1.038)	0.593			0.978(0.931-1.028)	0.389		
Sex		0.241				0.401		
Male	reference				reference			
Female	0.691(0.373-1.281)				0.703(0.308-1.601)			
Etiology		0.400				0.935		
Hepatitis B	1.147(0.378-1.474)				1.041(0.402-3.695)			
Others	reference				reference			
Child Pugh score		0.833				0.817		
5-6	0.864(0.599-1.587)				0.907(0.395-2.080)			
7-9	reference				reference			
Tumor subtype		0.083				0.169		
Perihilar cholangiocacinoma	reference				reference			
Distal cholangiocacinoma	0.713(0.487-1.045)				0.648(0.349-1.202)			
Differentiated histology		0.869				0.844		
poorly	reference				reference			
others	0.969(0.677-1.407)				0.948(0.557-1.613)			
TNM stage		0.673				0.611		
III	reference				reference			
IV	1.134(0.633-2.033)				1.240(0.541-2.841)			
Prior chemotherapy cycles		0.304				0.245		
≤6	Reference				reference			
*>*6	0.472(0.113-1.972)				0.415(0.094-1.830)			
Largest tumor size (cm)		0.079				0.390		
≤3	reference				reference			
*>*3	1.490(0.954-2.325)				2.940(1.054-5.201)			
CA199 (U/ml)		0.905				0.421		
>200	1.105(0.541-1.721)				1.403(0.616-3.196)			
≤200	reference				reference			
Extrahepatic metastases		0.629				0.519		
Yes	1.150(0.438-1.646)				1.242(0.299-1.839)			
No	reference				reference			
Ascites		0.755				0.490		
Yes	1.139(0.503-2.575)				1.472(0.491-4.418)			
No	reference				reference			
ECOG PS		0.816				0.445		
0-1	reference				reference			
1-2	1.132(0.514-1.690)				1.219(0.308-1.677)			

HR, hazard ratio; CI, confidence interval; ECOG, Eastern Cooperative Oncology Group; TNM, tumor node metastasis; CA199, carbohydrate antigen 199; PSM, propensity-matched; HAIC, hepatic artery infusion chemotherapy. The bold values denote statistical significance at P<0.05 level.

**Figure 5:**
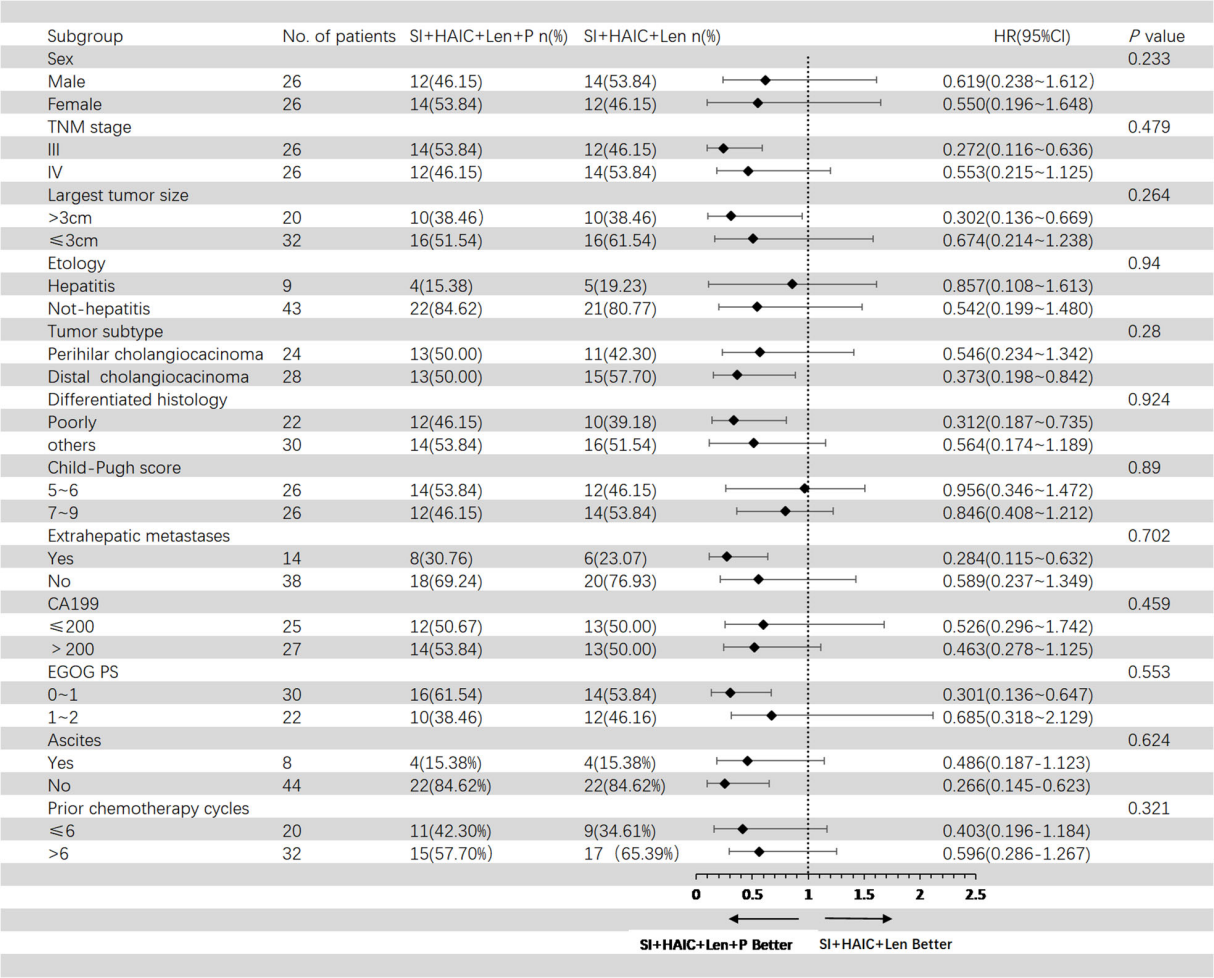
Forest plot of overall survival for subgroups in patients receiving the combination therapy of interventional treatment plus lenvatinib with or without PD-1 inhibitor. SI, biliary stenting implantation with iodine-125 seed strand; HAIC, hepatic artery infusion chemotherapy; ECOG, Eastern Cooperative Oncology Group; TNM, tumor node metastasis; CA199, carbohydrate antigen 199; PD-1, programmed death-1.

**Figure 6:**
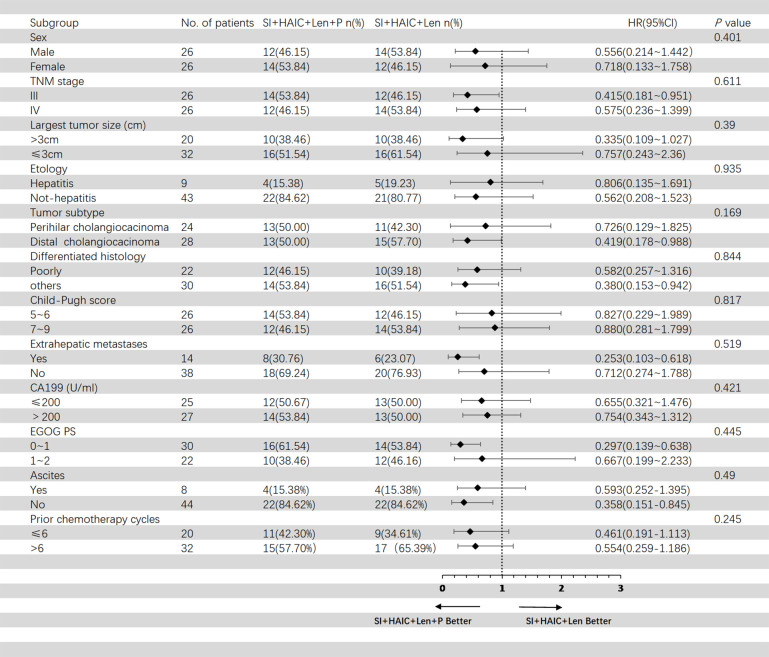
Forest plot of progression-free survival for subgroups in patients receiving the combination therapy of interventional treatment plus lenvatinib with or without PD-1 inhibitor. SI, biliary stenting implantation with iodine-125 seed strand; HAIC, hepatic artery infusion chemotherapy; ECOG, Eastern Cooperative Oncology Group; TNM, tumor node metastasis; CA199, carbohydrate antigen 199; PD-1, programmed death-1.

**Figure 5** showed the subgroup analysis for OS after PSM analysis. The following subgroups of patients showed a significant benefit in OS: TNM stage III, largest tumor size > 3cm, distal cholangiocarcinoma, poorly differentiated histology, extrahepatic metastases, ECOG PS 0-1; **Figure 6** showed the subgroup analysis for PFS after PSM analysis. The following subgroups of patients showed a significant benefit in PFS: TNM stage III, largest tumor size > 3cm, distal cholangiocarcinoma, extrahepatic metastases, ECOG PS 0-1.

### Treatment safety

No serious complications, such as acute liver failure, liver abscess, intraperitoneal bleeding, or radiation hepatitis, were observed as a result of the interventional treatment. The occurrence rates of common treatment-related adverse events (TRAEs), including fever, abdominal pain, and vomiting, were 15.38%, 57.69%, and 15.38% in the SI+HAIC+Len+P group, and 19.23%, 50.00%, and 23.07% in the SI+HAIC+Len group, respectively. There was no significant difference in TRAEs associated with interventional treatments between the two groups. All TRAEs were resolved following symptomatic treatment.

**Figure 7:**
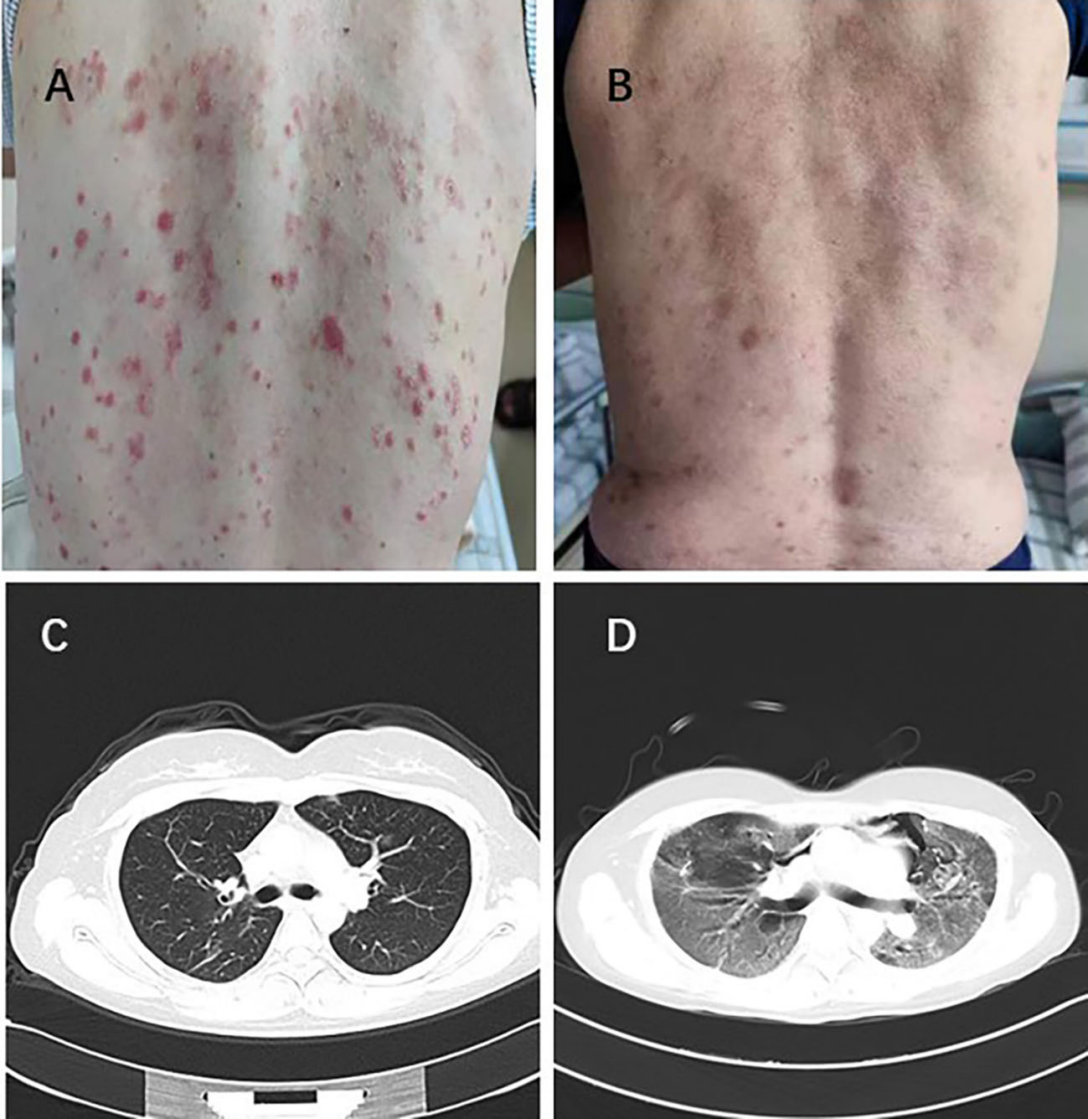
One patient experienced severe skin rash with a grade 4 **(A)** and recovered following lenvatinib reduction **(B)**. Lung imaging of another patient before immunotherapy **(C)**. The patient developed immune-related pneumonitis with a grade 4 after immunotherapy **(D)**.

**Table 5** showed all recorded adverse reactions associated with systemic therapy after PSM. The most common grade TRAEs were hypertension (8/26 30.76%), skin rash (6/26 23.07%), fatigue (15/26 57.69%), and diarrhea (6/26 23.07%) in SI+HAIC+Len+P group, consisted with that in SI+HAIC+Len group. 4 patients (15.38%) experienced grade 3–4 TRAEs in the SI+HAIC+Len+P group, while 2 patients (7.69%) in the SI+HAIC+Len group (*P* = 0.668). Among the SI+HAIC+Len+P group, 3 (11.53%) dose adjustments occurred due to skin rash (n = 1) (**Figures 7A, B**) and hypertension (n = 2) while 1 (3.84%) discontinuations of PD-1 inhibitor due to immune-associated pneumonitis (n = 1) (**Figures 7C, D**) were noted; respectively. Among the SI+HAIC+Len group, 2 (7.69%) dose reductions occurred due to skin rash (n = 1) and hypertension (n = 1) while 0(0.00%) discontinuations were noted. This triple combination therapy was generally safe and well-controlled in TRAEs.

**Table 5:** TRAEs in the study population (after PSM).

	All grades of TRAE			TRAE (grade > 3)		
	SI+HAIC+Len+P (n=26)	SI+HAIC+Len (n=26)	*P value*	SI+HAIC+Len+P (n=26)	SI+HAIC+Len (n=26)	*P value*
Fever, n (%)	4(15.38%)	5(19.23%)	0.714			
Decreased appetite, n (%)	8(30.76%)	9(34.61%)	0.768			
Abdominal pain, (%)	15(57.69%)	13(50.00%)	0.578			
Nausea/vomiting, no (%)	4(15.38%)	6(23.07%)	0.482			
Elevated serum AST or ALT, n (%)	10(38.46%)	9(34.61%)	0.773			
Thrombocytopenia, n (%)	5(19.23%)	4(15.38%)	1.000			
Gastrointestinal hemorrhage, n (%)	2(7.69%)	1(3.84%)	0.556			
Albumin decreased, n (%)	7(26.92%)	8(30.76%)	0.760			
Neutropenia, n (%)	6(23.07%)	7(26.92%)	0.749			
Creatinine increased, n (%)	12(46.15%)	11(42.30%)	0.780			
Liver abscess, n (%) Cholesteatoma, n (%)	1(3.84%) 2(7.69%)	1(3.84%) 3(4.05%)	1.000 0.532			
Cholecystitis, n (%)	1(3.84%)	2(7.69%)	1.000			
Hypertension, n (%)	8(30.76%)	6(23.07%)	0.532	2(7.69%)	1(3.84%)	0.556
Hand-foot skin reaction, n (%)	3(11.53%)	4(15.38%)	1.000			
Skin rash, n (%)	6(23.07%)	5(19.23%)	0.734	1(3.84%)	1(3.84%)	1.000
Proteinuria, n (%)	3(11.53%)	2(7.69%)	1.000			
Fatigue, n (%)	15(57.69%)	17(65.38%)	0.734			
Bleeding (gingiva), n (%)	2(7.69%)	1(3.84%)	1.000			
Diarrhea, n (%)	6(23.07%)	8(30.76%)	0.532			
Immune-associated pneumonitis	2(7.69%)	0(0.00%)	0.490	1(3.84%)	0(0.00%)	1.000
Immune-associated enteritis	1(3.84%)	0(0.00%)	1.000			
Immune-associated myocarditis	1(3.84%)	0(0.00%)	1.000			

AST, aspartate aminotransferase; ALT, alanine aminotransferase; PSM, propensity-matched; HAIC, hepatic artery infusion chemotherapy.

## Discussion

Our study investigated the efficacy and safety of triple therapy for ECC with MOJ. The main findings of our study were: 1) Compared to interventional therapy (SI+HAIC) plus Len, interventional therapy (SI+HAIC) combined with Len and PD-1 inhibitor had a significant improvement in DCR, OS, and PFS, showing a more favorable survival benefit. 2) The treatment options were significantly associated with OS and PFS; 3) The TRAEs were not significantly different between the groups.

The present study demonstrated that after biliary drainage and stent implantation, most patients had significantly reduced levels of serum total bilirubin (TBIL). Within 2 weeks of operation, the TBIL in the SI+HAIC+Len+P group significantly reduced by an average of 81.5%, compared to 80.8% in the SI+HAIC+Len group, indicating that the bile duct restoration made it possible to resume normal liver function. Consequently, the subsequent therapy for Len plus PD-1 inhibitor therapy could be used safely to inhibit tumor growth in our study. In this work, the results demonstrated a significant improvement in OS and PFS in the SI+HAIC+Len+P group compared to the SI+HAIC+Len group (median OS: 16.6 *vs*. 12.3 months, *P* = 0.001; median PFS 12.6 months *vs.* 8.5 months, *P* = 0.004); respectively, the SI+HAIC+Len+P group achieved greater DCR in comparison to the SI+HAIC+Len group (79.17% *vs.* 53.84%, *P* = 0.039). The result implied that the inclusion of an additional PD-1 inhibitor in the interventional therapy (SI+HAIC) combined with Len could potentially offer a more efficacious approach for the treatment of advanced ECC with MOJ, leading to improved survival outcomes. Previous studies showed that adding local-regional therapy (51.6% radiotherapy, 9.7% HAIC, 19.4% TACE) to toripalimab and Len yielded confirmed response rates (32%), consistent with ORR (34.61%) in the SI+HAIC+Len+P group in our study (20); A recent study by Wang et al. (21) reported radiotherapy combined with Len and PD-1 inhibitors therapy for unresectable cholangiocarcinoma with a median OS of 11.7 months, a median PFS of 7.9 months and ORR of 32.3%, while the present study reported higher median PFS of 12.6 months, median OS of 16.6 months and ORR of 34.61% in the SI+HAIC+Len+P group. It could be due to the additional effect of HAIC therapy in our study and this published study included more liver metastatic sites (83.9%) associated with poor prognosis.

In comparison to the SI+HAIC+Len group, the inclusion of PD-1 inhibitor therapy in the SI+HAIC+Len+P group resulted in a notable enhancement in survival rates among patients with ECC and MOJ. Our findings underscore the importance of incorporating PD-1 inhibitor therapy as an additional treatment modality for ECC patients with MOJ, as it has the potential to further augment the therapeutic efficacy of SI+HAIC+Len treatment. One significant histological characteristic of cholangiocarcinoma is the presence of a dense desmoplastic stroma containing cancer-associated fibroblasts, which frequently envelop the tumor cells. Studies have indicated that the fibrotic tumor microenvironment, in conjunction with the infiltration of innate immune cells like myeloid-derived suppressor cells (MDSCs) and tumor-associated macrophages (TAMs), contributes to the establishment of an immunosuppressive tumor immune microenvironment (TIME) in cholangiocarcinoma (22, 23). the infiltration of Tregs is associated with a poor prognosis in patients with cholangiocarcinoma (24). The T-cell infiltration and surface marker expression in cholangiocarcinoma hold significant mechanistic and therapeutic implications for immune checkpoint inhibitors. Cancer cells have been observed to upregulate PD-L1 as a means to evade T-cell attack through the PD-L1/PD1 axis, leading to the induction of apoptosis in tumor-infiltrating lymphocytes (TILs). This elevated PD-L1 expression is associated with unfavorable OS (25). The presence of the PD1/PD-L1 axis has been observed in cholangiocarcinoma tumor cells and TILs, indicating the possibility of eliciting immune responses through anti-PD1 or anti-PD-L1 immunotherapy (26). However, the efficacy of immunotherapy alone remains limited, many clinical trials have attempted to combine immunotherapy with chemotherapy, locoregional therapy, or targeted therapies to improve response rates. The observed effectiveness of the triple therapy in our study can be attributed to these discoveries: The activation of tumor-specific antigens and the improvement of clinical effectiveness of PD-1 antibodies are facilitated by HAIC; Additionally, HAIC induces the elimination of tumor cells, leading to the entry of non-proliferative cells into the proliferative phase and synchronization of the tumor cell cycle (27). This synchronization proves beneficial for the γ-rays emitted by iodine-125 particles in targeting cycle-sensitive cells for destruction (28). Vascular endothelial cells possess robust proliferative capacity and exhibit susceptibility to radiation (29). Following the introduction of iodine-125 radioactive particles, radiation induces direct harm to vascular endothelial cells, leading to diminished expression of VEGF and subsequent reduction in angiogenesis (30). Consequently, the formation of tumor neovascularization is impeded. Simultaneously, the TKI exhibits the ability to impede the development of tumor neovascularization and induce the upregulation of PD-L, thereby facilitating immune cell infiltration into the tumor (31, 32). This phenomenon enhances the clinical efficacy of HAIC and PD-1 antibodies. PD-1 inhibitors function by obstructing the signals on T cells, thereby promoting an anti-tumor immune response (33). However, it is imperative to validate this conclusion through additional experimental investigations (34, 35).

Based on subgroup analyses of TNM stage III, largest tumor size > 3cm, distal cholangiocarcinoma, poorly differentiated histology, extrahepatic metastases, ECOG PS 0-1, a better OS was achieved by the SI+HAIC+Len+P group, while the other subgroups not. This may be explained by insufficient sample size and the use of PD-1 inhibitor; Additionally, the multivariate analysis showed the therapy option was independently associated with OS and PFS. Thus, the results of our study suggested that PD-1 inhibitors are potentially beneficial in improving clinical outcomes for these ECC patients.

TRAEs were acceptable in this study. The SI+HAIC+Len +P group had a higher prevalence of TRAEs than the SI+HAIC+Len group. Despite this, no significant differences were observed between the groups. Symptoms or dose adjustments were able to alleviate or eliminate these TRAEs, which were mostly grade 1 or 2. The combining interventional therapy and Len with/without PD-1 inhibitor in the present study resulted in similar adverse events as reported in previous studies. Neither toxic effects nor safety signals were observed. Thus, the SI+HAIC+Len+P therapy was both feasible and acceptable for these patients.

This study has several limitations. First, it is a retrospective study on a small sample of patients, which reduces the ability to make general conclusions. Second, in our study, tislelizumab and sintilimab were used as PD-1 inhibitors primarily due to their availability through complementary drug policies or health insurance. Furthermore, compared to nivolumab and toripalimab which were widely used for ECC, patients faced much fewer financial burdens. Additionally, these two PD-1 inhibitors have been proven to be effective and safe in advanced ECC trials (36, 37). Third, we did not further evaluate microsatellite instability-high status, mismatch repair protein deficiency, and programmed death-ligand 1 before using the PD-1 inhibitor.

In conclusion, for ECC patients with MOJ, interventional therapy (SI+HAIC) combined with Len and PD-1 inhibitor may result in a better OS and PFS and it deserves consideration as an optimization strategy.

## Data availability statement

The original contributions presented in the study are included in the article/**Supplementary Material**. Further inquiries can be directed to the corresponding authors.

## Ethics statement

The studies involving humans were approved by the Ethics Committee of Fujian Medical University Union Hospital, Fuzhou, China. The studies were conducted in accordance with the local legislation and institutional requirements. Written informed consent for participation was not required from the participants or the participants’ legal guardians/next of kin in accordance with the national legislation and institutional requirements. Written informed consent was obtained from the individual(s) for the publication of any potentially identifiable images or data included in this article.

## Author contributions

L-WL: Project administration, Software, Supervision, Writing – original draft. KK: Formal analysis, Project administration, Writing – review & editing. W-ZY: Formal analysis, Investigation, Writing – review & editing. RC: Writing – original draft. NH: Conceptualization, Writing – review & editing. Z-ZW: Conceptualization, Writing – review & editing.
