# Supplementary figures and images for: Safety and efficacy of biliary stenting combined with iodine-125 seed strand followed by hepatic artery infusion chemotherapy plus lenvatinib with PD-1 inhibitor for the treatment of extrahepatic cholangiocarcinoma with malignant obstructive jaundice
Source: Front Immunol. 2024 Jan 15;14:1286771. doi: 10.3389/fimmu.2023.1286771 (PMC10822914; doi:10.3389/fimmu.2023.1286771)

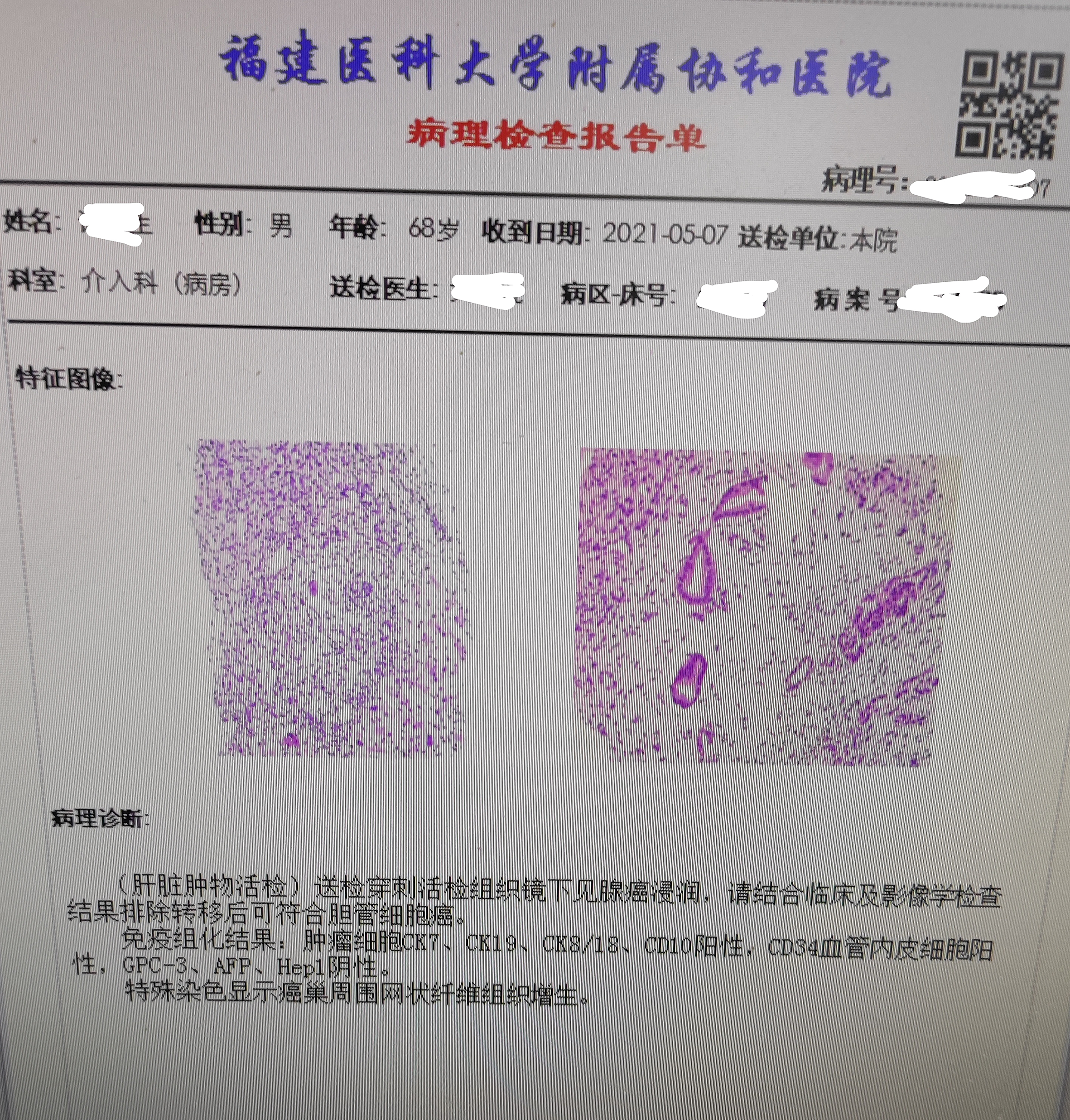

Supplement: Supplementary file 1 [file Image_1.tif]

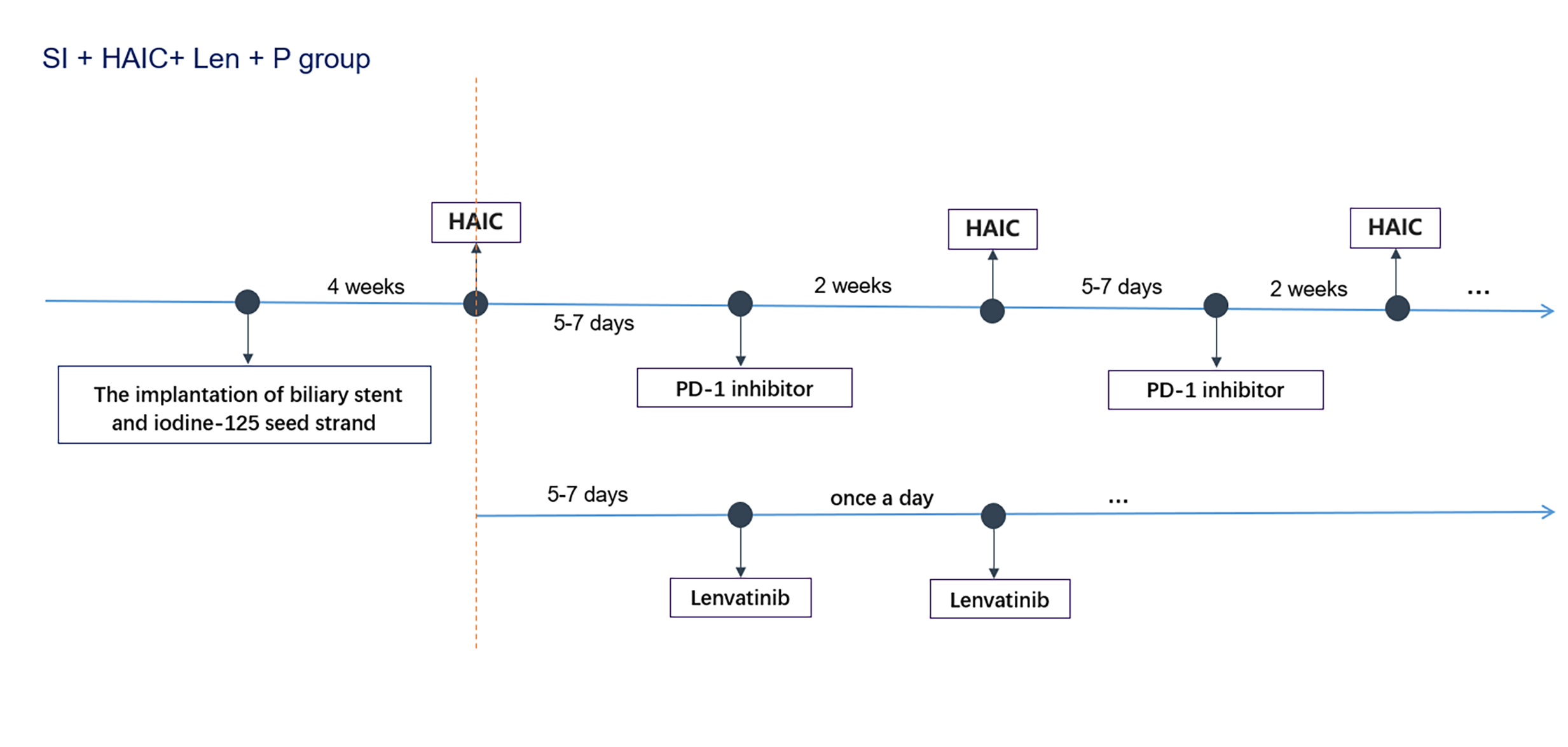

Supplement: Supplementary file 2 [file Image_2.tif]

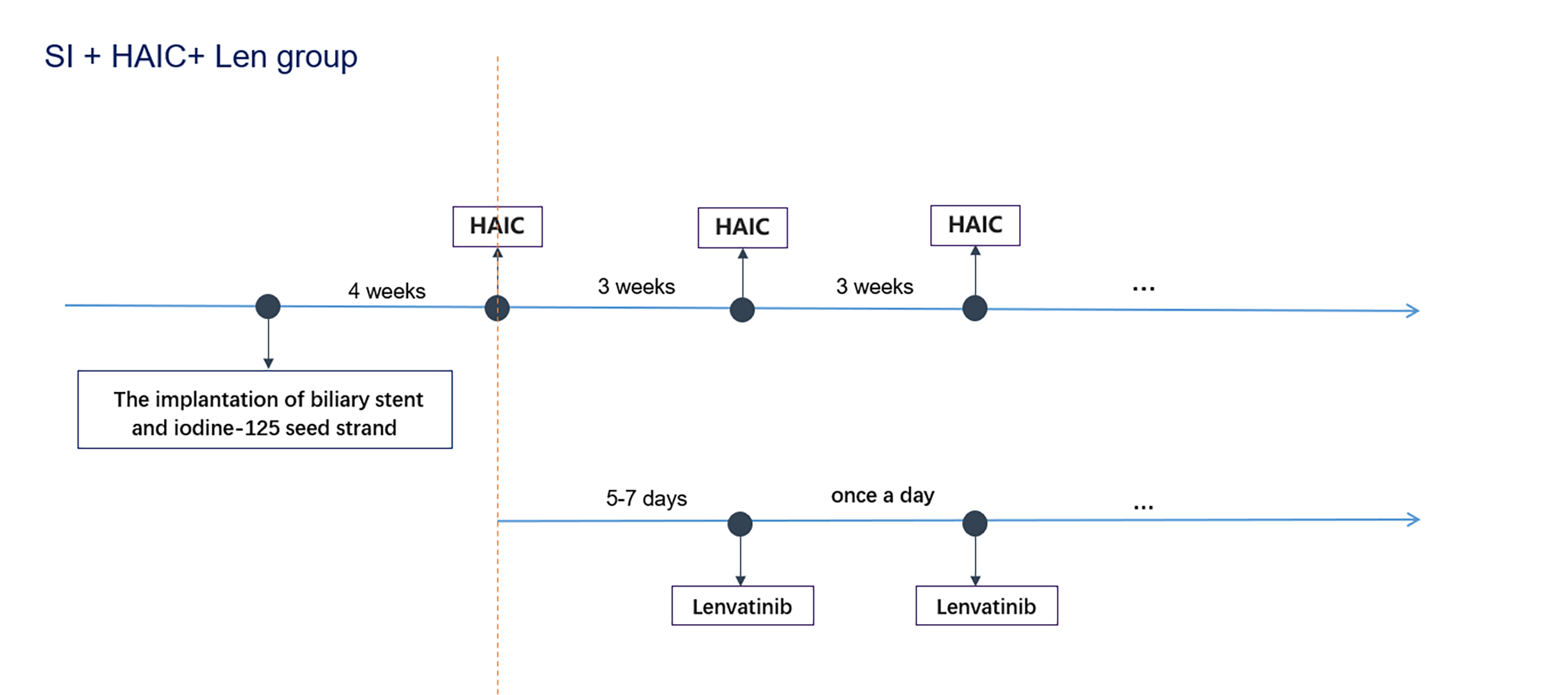

Supplement: Supplementary file 3 [file Image_3.tif]
